# Prevalence Rates of Abdominal Obesity, High Waist-to-Height Ratio and Excess Adiposity, and Their Associated Cardio-Kidney-Metabolic Factors: SIMETAP-AO Study

**DOI:** 10.3390/nu16223948

**Published:** 2024-11-19

**Authors:** Antonio Ruiz-García, Adalberto Serrano-Cumplido, Carlos Escobar-Cervantes, Ezequiel Arranz-Martínez, Vicente Pallarés-Carratalá

**Affiliations:** 1 Lipids and Cardiovascular Prevention Unit, Pinto University Health Centre, 28320 Madrid, Spain; aruizg@salud.madrid.org; 2 Department of Medicine, European University of Madrid, 28005 Madrid, Spain; 3 Repelega Health Centre, 48920 Bizkaia, Spain; adalberto.serrano@semergen.es; 4 Department of Cardiology, La Paz University Hospital, 28046 Madrid, Spain; 5 San Blas Health Centre, 28981 Madrid, Spain; ezequiel.arranz@salud.madrid.org; 6 Department of Medicine, Jaume I University, 12006 Castellon, Spain; pallarev@uji.es

**Keywords:** abdominal obesity, adiposity, adults, cardiovascular risk factors, prevalence, waist-to-height ratio

## Abstract

Background/objective: In addition to obesity, adiposity and abdominal obesity (AO) are parameters included in the cardiovascular–kidney–metabolic (CKM) syndrome. However, their prevalence and association with the other CKM factors have been less studied. Our study aimed to determine the prevalence rates of AO, high waist-to-height ratio (WtHR), and excess adiposity (EA), and to compare their associations with CKM factors. Methods: A cross-sectional observational study was conducted with a random population-based sample of 6,588 study subjects between 18 and 102 years of age. Crude and sex- and age-adjusted prevalence rates of AO, high-WtHR, and EA were calculated, and their associations with CKM variables were assessed by bivariate and multivariate analyses. Results: The adjusted prevalence rates for AO, high-WtHR, and EA were 39.6% (33.6% in men; 44.9% in women), 30.6% (31.1% in men; 30.6% in women), and 65.6% (65.6% in men; 65.3% in women), respectively, and they increased with age. The main independent factors associated with AO, high-WtHR, and EA were hypertension, diabetes, prediabetes, low HDL-C, hypercholesterolaemia, hypertriglyceridemia, physical inactivity, hyperuricemia, and chronic kidney disease. Conclusions: Two-thirds of the adult population have EA, one-third have AO, and one-third have high-WtHR. These findings support that the other factors of CKM syndrome, in addition to hyperuricemia and physical inactivity, show an independent association with these adiposity-related variables.

## 1. Background

The prevalence of obesity in adults continues to increase worldwide, rising to 18.5% among women and 14.0% among men in 2022 (vs. 8.8% and 4.8%, respectively, in 1990), while in children and adolescents, it reached 6.9% in women and 9.3% in men (vs. 1.7% and 2.1%, respectively, in 1990) [1]. The latest annual report from the Spanish National Health System stated that the prevalence of obesity in adults was 16.0%, and 10.3% in subjects between 2 and 17 years of age [2]. Similar or more worrying are the prevalence rates shown by the recent ENE-COVD study [3] in the Spanish population between 2 and 17 years of age, reaching 30.0% overweight (33.7% in men and 26.0% in women) and 10.7% obesity (13.4% in men and 7.9% in women).

Obesity is a chronic metabolic disorder universally measured by the body mass index (BMI), which the World Health Organization defines as a disease in which the accumulation of body fat and its redistribution harms the health of the affected people [4]. However, why do we continue to say obesity when we mean adiposity? The main criticisms about BMI are that it incorrectly classifies people with significant muscle mass as obese, since it does not distinguish between fat and muscle mass, and does not provide information about the body fat distribution. Although the main characteristic of obesity is an abnormal or excessive accumulation of body fat, BMI does not encompass the complex biology of excess adiposity (EA) and does not assess this alteration [5,6]. Body fat distribution appears to be a better predictor of comorbidity risk than obesity because the quantity of body fat mass may correlate with certain adverse clinical outcomes or adiposity-based complications, but is not adequately reflected by BMI [5,6].

There are many indices to assess body composition [7], such as abdominal obesity (AO), waist-to-height ratio (WtHR), waist-to-hip ratio, a body shape index (ABSI) [8], Conicity Index [9], anthropometric risk index (ARI) [10], body roundness index (BRI) [11], body adiposity index (BAI) [12], and visceral adiposity index (VAI) [13], which are based on weight, height, BMI, waist circumference (WC), hip circumference, ABSI, and even HDL-C and triglyceride levels. Unlike these indicators, the CUN-BAE (according to its acronym in Spanish, *Clínica Universitaria de Navarra*—Body Adiposity Estimator) is based on BMI, age, and sex [14]. To better evaluate obesity, the American Association of Clinical Endocrinology defined a new diagnostic term for obesity as an adiposity-based chronic disease that alludes to a precise pathophysiological basis and avoids the confusion related to the multiple meanings of the term obesity, and recommended the use of anthropometric indicators that are more closely related to adiposity [15,16].

Adiposity is positively correlated with cardio-metabolic diseases, especially when the excess fat accumulates as visceral adipose tissue (VAT) [17,18]. Its presence is the preamble for the appearance of neoplastic, cardiovascular, hepatic-biliary, respiratory, and musculoskeletal diseases, as well as type 2 diabetes mellitus (T2DM) [19,20]. The limitations of BMI in detecting fat accumulation or its redistribution in the body could relegate its use to obesity screening, leading to focus on adiposity by determining the percentage of body fat or VAT accumulation [18,21].

Cardiovascular–kidney–metabolic (CKM) syndrome is a systemic disorder attributable to pathophysiological interactions among the cardiovascular system, chronic kidney disease (CKD), and other cardiometabolic risk factors, such as EA, obesity, AO, prediabetes, T2DM, hypertension, hypertriglyceridemia, and metabolic syndrome (MetS) [22]. CKM syndrome is partially explained by genetic inheritance and is influenced by lifestyle and psychosocial conditions, so dysfunction and fat accumulation usually begin during the early stages of life. The conjunction of these factors progressively produces proinflammatory states, oxidative stress, and insulin resistance, followed by subclinical stages that, if they persist, develop into CKM syndrome. Knowing the prevalence of the causal factor of EA and other related variables such as high-WtHR and AO, in addition to the clinical factors and disorders that are independently related to these variables, and having tools that allow for early diagnosis, will allow appropriate actions to be taken to improve clinical benefits.

There are many studies that determine the prevalence rates of overweight and obesity; however, we did not find any Spanish population study that evaluated the prevalence rates of variables related to adiposity from 18 to over 100 years of age., nor which cardiovascular, renal or metabolic factors are most strongly related to AO, high-WtHR, and EA. On the other hand, despite the existence of so many adiposity assessment indices, there is a gray area in obesity–adiposity research that may explain many of the difficult-to-understand phenomena in this field. AO, high-WtHR, and EA are parameters that increase the risk of cardiometabolic disorders and all-cause mortality, and that assess body fat distribution [23,24]. Therefore, our study aimed to determine the prevalence rates of these parameters in adults, and to compare their associations with cardiovascular, renal, and metabolic factors.

## 2. Materials and Methods

### 2.1. Study Design

We conducted a cross-sectional observational multicentre sub-study from the SIMETAP study, whose design and methodology have been previously published [25]. This study was authorized by the Madrid Health Service (SERMAS, according to its acronym in Spanish *Servicio Madrileño de Salud*). The healthcare of 99% of the census population (5,144,860 adults) of the Region of Madrid (Spain) was provided in 260 primary healthcare centres of SERMAS, through their Health Identity Cards (HICs). Briefly, a simple random sampling was performed using MS Excel’s randbetween function on HICs, assigned to the research physicians (194,073 adults), until the necessary sample size was reached to evaluate the study aims (response rate 62.9%). One hundred and twenty-one physicians from sixty-four healthcare centres (Figure S1, Supplementary Materials) collected data and conducted interviews with participants. Inclusion criteria: Adults with informed consent and the clinical and laboratory data necessary to be assessed. Patients with terminal illnesses or cognitive impairment, dementia, schizophrenia, moderate or severe psychosis, residents of nursing homes, pregnant women, or people who were participating in other clinical studies were excluded from this study according to the protocol approved by the Research Ethics Committee (Figure S1, Supplementary Materials). All information assessed in the study were collected from the primary care electronic health records in a real-world data setting.

### 2.2. Assessment Variables

The primary endpoints of the study were AO, high-WtHR, and AE. Women with a WC ≥ 88 cm and men with a WC ≥ 102 cm were considered AO, according to the criterion included in the definition of metabolic syndrome (MetS) for the European population [26]. WtHR was calculated as the WC measurement divided by height measurement, in centimetres. The cut-off point to consider a high-WtHR was ≥0.60 for both genders [27]. The CUN-BAE body fat indices > 25% for men and >35% for women were considered to be EA [14]. The criteria and definitions of the other comorbidities or medical conditions assessed are reported in detail in Table S1, Supplementary Materials.

### 2.3. Statistical Analysis

Mean, standard deviation (SD), median, and interquartile range (IQR) of age and anthropometric parameters were determined. Qualitative variables were described by the number and percentage of each category. Prevalence rates were determined for the overall adult study population and according to age groups. The age- and sex-adjusted prevalence rates were calculated by the direct method, according to the population data from the Spanish National Institute of Statistics. Percentages and odds ratios (ORs) were used with a 95% confidence interval (CI). Comparison of percentages was performed using the Chi-squared test or Fisher’s exact test when at least 20% of the expected frequencies were less than five. A Shapiro–Wilk test was used to check the data fitting to normal distribution for continuous variables. If the variables showed normal distribution, they were compared using Student’s *t*-test or analysis of variance. Cohen’s *d* was used to assess the effect size of standardized mean differences, according to the proximity to the following absolute *d*-values: 0.2 small; 0.5 medium; and 0.8 large. The correlations among the WC, WtHR, CUN-BAE, and age were assessed using Pearson’s correlation *ρ* coefficient, defining the strength of the correlations as null (0.0 < 0.1), low (0.1 < 0.3), medium (0.3 < 0.5), high (0.5 < 0.7), and very high (0.7 < 1.0). Multivariate logistic regression analyses were performed using the backward stepwise method to assess the individual effect of comorbidities and clinical conditions on the dependent variables (AO, high-WtHR, EA). All variables that had an association in the bivariate analysis up to a *p* value < 0.10 were entered into the model, except for those variables that could bias the analysis such as overweight and obesity, or that included other parameters evaluated individually such as MetS [26] and fatty liver index (FLI) [28], and for erectile dysfunction, because it affects only men. Subsequently, the variable that less contributed to the fit of the analysis was eliminated at each step. All statistical testing were two-tailed, with *p*-value < 0.05 used to determine statistical significance. All analyses were performed with SPSS Statistics for Windows, version 25 (IBM Corporation, Armonk, NY, USA).

## 3. Results

### 3.1. Study Population

Our study included 6588 people (55.9% women) that ranged from 18.0 to 102.8 years of age. Their mean (SD) age and median (IQR) were 55.1 (17.5) years and 54.7 (41.7–68.1) years, respectively. The median (IQR) ages of the male and female populations were 55.0 (42.4–67.5) years and 54.5 (41.0–68.8) years, respectively, with a non-significant difference in mean [SD] ages between men (55.3 [16.9] years) and women (55.0 [18.0] years) (*p* = 0.634). Female percentages among people with AO, high-WtHR, and EA were 62.3%, 54.7%, and 54.7%, respectively. The means (SD) of the anthropometric parameters of the study population were similar to their respective medians (IQR), although they showed significant differences between the male and female population, except for BMI and WtHR (Table 1). 

The means (SD) of anthropometric parameters in populations with AO, high-WtHR, and EA were similar to their respective medians (IQR), although significant differences were found between them, except for weight between populations with AO and with high-WtHR (Table S2, Supplementary Materials). The percentiles for cut-off measures used to diagnose AO (≥102 cm in men; ≥88 cm in women) were p65 and p50, respectively. The percentiles of the cut-off point 0.60 to estimate high-WtHR in our study were the same in the overall population as well as in men and women (p65). The percentiles for the cut-off point 0.50 were p22 in the overall population (p15 in male; p28 in female), and p45 for the cut-off point 0.55 in the overall population (p40 in male; p57 in female). The percentiles for the CUN-BAE body fat index cut-off points used to estimate EA (>25% for men; >35% for women) were p25 and p30, respectively. The strength of correlation between age and the parameters WC, WtHR, and CUN-BAE in people with AO, high-WtHR, and EA were null, low, and very low in men, respectively, and low, null, or medium in women, respectively (Figure S2, Supplementary Materials).

The highest correlation among the anthropometric variables was between WC and WtHR, both in the general population (*ρ* = 0.9), as well as in the populations with AO (*ρ* = 0.8), with high-WtHR (*ρ* = 0.7), and with EA (*ρ* = 0.9) (*p* < 0.001). The strength of the correlation between WC and CUN-BAE is moderate (*ρ* = 0.4) (*p* < 0.001) in the general population, null (*ρ* = 0.1) in the populations with AO or with high-WtHR (*p* < 0.001), and low (*ρ* = 0.2) in the population with EA (*p* < 0.001). The strength of the correlation between WtHR and CUN-BAE is high (*ρ* = 0.7) (*p* < 0.001) in the general population, and medium (*ρ* = 0.5) in the AO, high-WtHR, and EA populations (*p* < 0.001) (Table S3, Supplementary Materials).

### 3.2. Prevalence Rates

**Figure 1:**
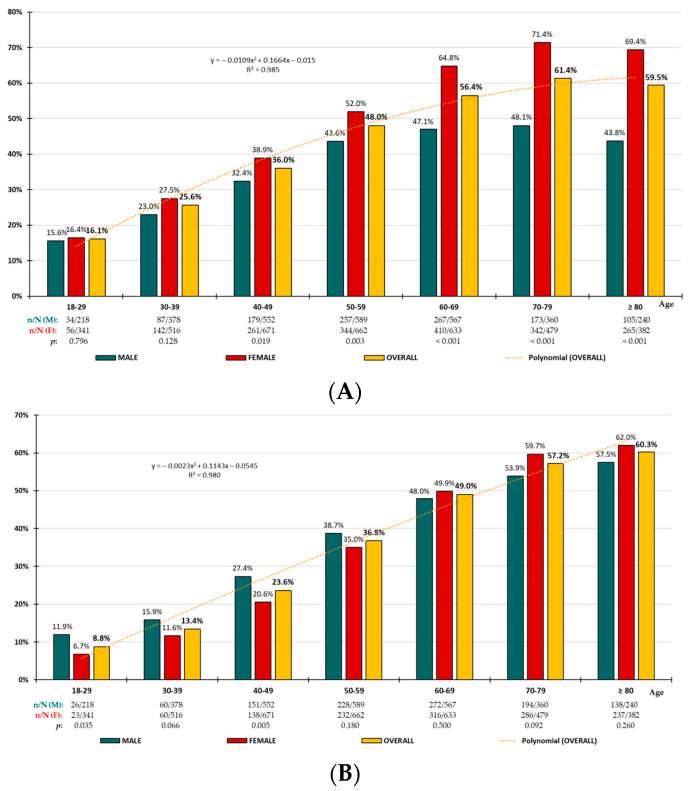

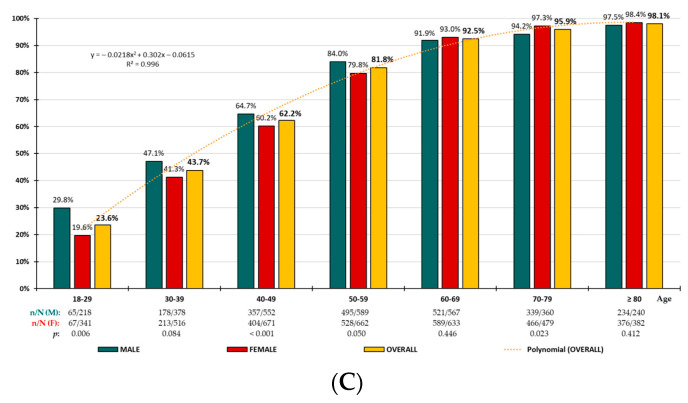
Age-specific prevalence rates of abdominal obesity (**A**), high-WtHR (**B**), and excess adiposity (**C**); n: number of cases; N: sample size; M: male; F: female; *p*: *p*-value of the difference in percentages (M vs. F).

Crude and adjusted prevalence rates are shown in Table 2. The difference in prevalence rates between men and women was significant for people with AO, being higher in men for all age groups except for those under 40 years of age. It was non-significant for people with high-WtHR, except for those aged under 30 years and between 40 and 49 years of age, and it was non-significant for people with EA, except for those aged under 30 years, between 40 and 49 years of age, and between 70 and 79 years of age. The distribution of AO, high-WtHR, and EA prevalence rates by age groups increased precisely (R^2^ > 0.98) with age according to polynomial functions (Figure 1).

### 3.3. Analysis for Populations with and Without AO, High-WtHR, and EA

All clinical variables were significantly higher (*p* < 0.001) in populations with AO, high-WtHR, and EA than in populations with non-AO, non-high-WtHR, and non-EA, respectively, except for high-density lipoprotein cholesterol (HDL-c) and estimated glomerular filtration rate, which were significantly higher in non-AO, non-high-WtHR, and non-EA populations. Differences in aspartate aminotransferase were non-significant between the three comparison groups, nor creatinine between populations with and without AO or high-WtHR, nor low-density lipoprotein cholesterol between populations with and without high-WtHR (Table 3). 

**Figure 2:**
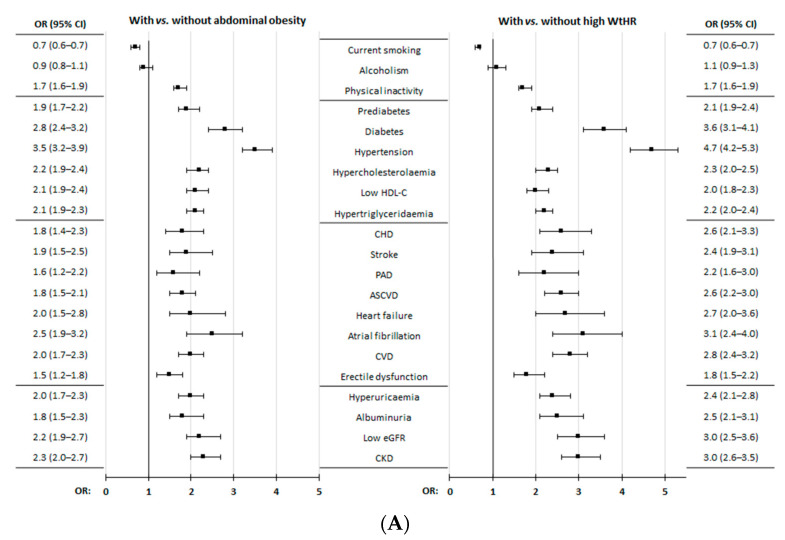

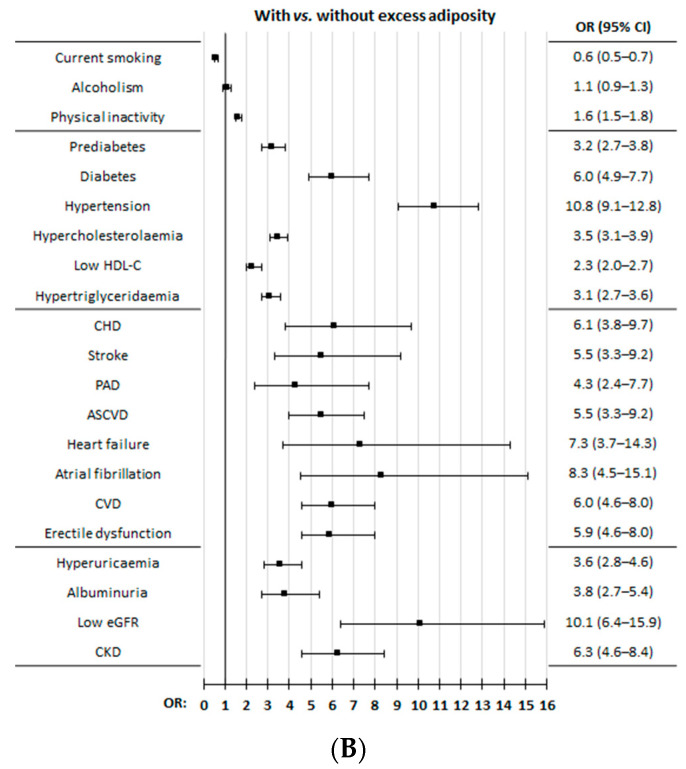
(**A**) Comorbidities and medical conditions in populations with and without abdominal obesity and with and without high-WtHR. (**B**) Comorbidities and medical conditions in populations with and without excess adiposity. ASCVD: atherosclerotic cardiovascular disease; CHD: coronary heart disease; CI: confidence interval; CKD: chronic kidney disease; CVD: cardiovascular disease; eGFR: estimated glomerular filtration rate; HDL-C: high-density lipoprotein cholesterol; OR: odds ratio; PAD: peripheral arterial disease. The definitions of comorbidities or medical conditions are shown in Table S1 (Supplementary Materials).

All the comorbidities and clinical conditions showed a significant association (*p* < 0.001) with AO, high-WtHR, and EA, except alcoholism. Overweight was similar in the AO population than in the non-AO population (*p* = 0.477), and participants who were currently smoking were significantly higher (*p* < 0.001) in non-AO, non-high-WtHR, and non-EA populations (Figure 2; Table S4, Supplementary Materials).

The percentage of the population at moderate- to high-risk of CKD was 14.4% in adults with AO, 16.5%% in those with high-WtHR, and 12.7% if they had EA (ORs of 2.3, 2.6 and 5.3, respectively). The percentage of the population at high or very high cardiovascular risk was 60.7% in adults with AO, 68.0% in those with high-WtHR, and 57.9% if they had EA (ORs of 2.8, 4.1 and 7.8, respectively) (Table S3, Supplementary Materials).

**Figure 3:**
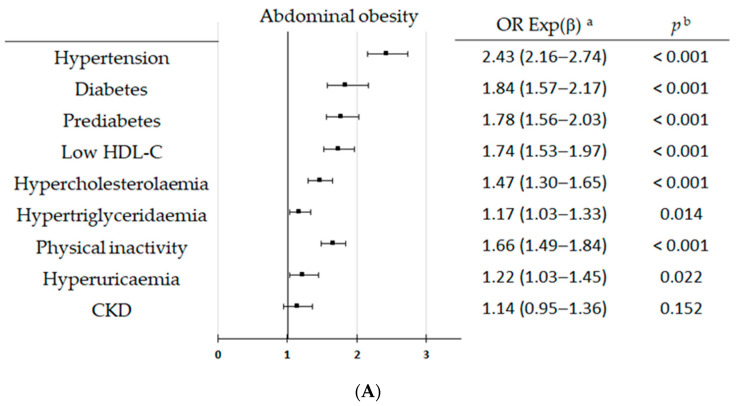

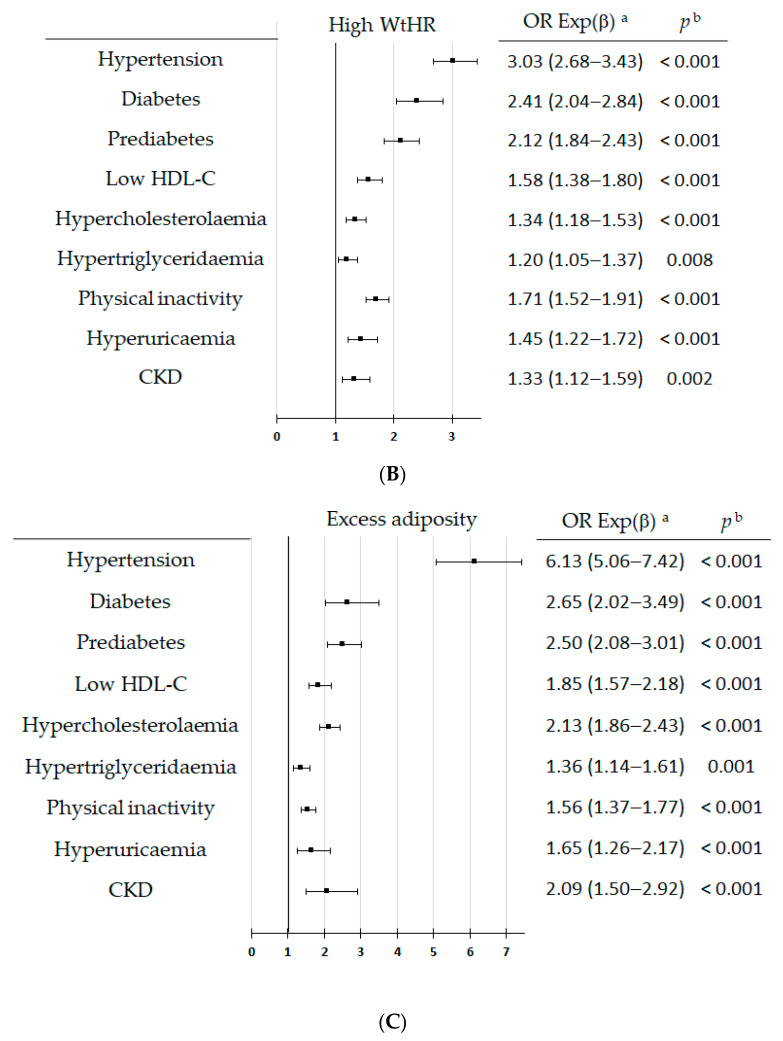
Multivariate analysis of factors and medical conditions for abdominal obesity (**A**), high-WtHR (**B**), and excess adiposity (**C**). CKD: chronic kidney disease; HDL-C: high-density lipoprotein cholesterol; WtHR: waist-to-height ratio. Definitions of the clinical conditions are shown in Table S1 (Supplementary Materials). ^a^ OR Exp (β): odds-ratio (95% confidence interval); ^b^ *p*: *p*-value of Wald test with one degree of freedom. CKD: chronic kidney disease; HDL-C: high-density lipoprotein cholesterol. Definitions of the clinical conditions are shown in Table S1 (Supplementary Materials). ^a^ OR Exp (β): odds-ratio (95% confidence interval); ^b^ *p*: *p*-value of Wald test with one degree of freedom.

Multivariate analysis showed that hypertension, prediabetes, diabetes (DM), low HDL-C, hypercholesterolemia, hypertriglyceridemia, physical inactivity, hyperuricemia, and CKD were associated with OA, high-WtHR, and EA, except for CKD, which did not show a significant association with OA (*p* = 0.152) (Figure 3 and Table S4, Supplementary Materials).

## 4. Discussion

### 4.1. Prevalence Rates

Our results showed that assessing BMI alone to determine obesity prevalence may underestimate the main characteristic of obesity, which is the abnormal or excessive accumulation of body fat, since BMI does not assess this alteration, and therefore, other anthropometric parameters should be considered. In our previous SIMETAP-OB study [29] conducted with the same population, we showed that the adjusted prevalence rates of obesity were 25.0% (26.2% in men; 24.5% in women, respectively). However, the adjusted prevalence rates of AO, high-WtHR, and EA of the present study were higher (39.6%, 30.6%, and 65.6%, respectively). The ENRICA study [30] showed results closer to our data for AO prevalence in the Spanish adult population (35.5%), with the percentages also being significantly higher in women than in men in almost all age groups. The 2018 National Health and Nutrition Examination Survey [31] conducted in the United States among the overall population showed an AO prevalence of 53.1% (43.2% in men; 62.9% in women). This higher prevalence could be justified because the WC cut-off point for estimating AO was lower in men (100 cm) than that considered in Europe.

The NICE guidelines [27] state that BMI should be interpreted with caution, as it is not a direct measure of central adiposity and highlight that the WtHR assessment should be encouraged in adults with a BMI < 35 kg/m^2^ as it provides a more accurate estimate of central adiposity. A WtHR ≥ 0.6 indicates an increased risk of hypertension, DM, and cardiovascular disease (CVD). From the published data of the ENPE study [32], it can be deduced that the prevalence of high-WtHR in the Spanish adult population was 73.2%, a percentage that is double that of the results of our study, most likely because the cut-off point they used to estimate a high-WtHR was only 0.50. The percentiles for high-WtHR using the cut-off point 0.50 were very low in our study (p22 in the general population [p15 in men; p28 in women]), which would have meant that around 75% of our study population would have a high-WtHR if the cut-off point 0.50 had been used. In contrast, the percentiles for high-WtHR using the 0.60 cut-off point of our study were p65 in the general population and similar for both men and women. Our study showed that 99.3% of subjects with high-WtHR had EA, but 58.9% of subjects with WtHR < 0.60 also had EA. Similarly, subjects with or without AO had EA in 97.9% and 53.8%, respectively. High-WtHR had high specificity (99%) and low sensitivity (48.5%) to identify EA calculated according to the CUN-BAE body fat index [14]. The EA determined by the CUN-BAE is associated with an increased risk of DM and CVD [14]. The percentiles corresponding to the CUN-BAE body fat index cut-off points used to estimate EA (>25% for men; >35% for women) were low in our study population (p25 and p30, respectively). On the other hand, the correlation matrices showed novel and intriguing findings for the subpopulations studied, as the correlations of CUN-BAE with WC was greatly reduced when AO or high-WtHR was present, and to a lesser extent, when there was EA (Table S3, Supplementary Materials).

### 4.2. Effect of CKM Factors and Clinical Conditions on AO, High-WtHR, and EA

The construct of CKM syndrome is based on the factors included in the first two stages [22]. Stage 1 includes individuals with excess adipose tissue as identified by overweight, obesity, and AO, or with dysfunctional adipose tissue as reflected by impaired glucose tolerance and hyperglycaemia. Stage 2 includes individuals with metabolic risk factors (hypertriglyceridemia, hypertension, MetS, or T2DM) and moderate- or high-risk CKD.

A prospective cohort analysis of the PREDIMED study [33] that included 7144 people aged 55 to 80 years free of CVD but with T2DM or high risk of CVD showed an inverse association between physical activity intensity and AO. Our study showed that physical inactivity was an independent major factor associated with AO, high-WtHR, and EA, whose ORs were 1.7, 1.7, and 1.6, respectively.

The ILERVAS project [34] carried out with 8188 overweight middle-aged subjects, without T2DM, CVD or CKD, showed a weak association between prediabetes and the equations used to estimate body composition, with no obesity index appearing to be the perfect biomarker to detect individuals with prediabetes or that could replace BMI in routine clinical practice. The multivariate analysis performed in our previous SIMETAP-PRED study [35] showed similar results, since obesity was an independent factor associated with prediabetes with an OR of 1.7, while increased WtHR was associated only with an OR of 1.3. The PREDAPS study included 2022 participants aged 30 to 74 years with prediabetes or with unaltered glucose metabolism and showed that prediabetes was associated with AO (OR 2.7) and with an increased WtHR ≥ 0.55 (OR 2.8) [36]. Other studies [37,38] showed that both WC and WtHR are better than BMI for detecting DM. The multivariate analysis performed in our study with the overall adult population data showed that prediabetes was a major independent factor associated with AO (OR 1.8), high-WtHR (OR 2.1), and EA (OR 2.5), and that DM was the second strongest major independent factor associated with AO (OR 1.8), high-WtHR (OR 2.4), and EA (OR 2.7).

Our results also showed that the diagnostic criteria for MetS [26] were associated with the assessed adiposity-related variables (Table S3, Supplementary Materials), supporting that AO was the main condition for MetS. The PREDAPS study [36] showed that hypertension, hypertriglyceridemia, and low HDL-c were associated with AO (ORs 2.8, 2.7, and 2.3, respectively) and with increased WtHR (ORs 3.2, 2.1, and 2.1, respectively). VAT accumulation is strongly associated with the hypertension development [39]. Our study supported that hypertension was the first strongest major independent factor associated with AO, high-WtHR, and EA (ORs 2.4, 3.0, and 6.1, respectively), that low HDL-c was a major independent factor associated with AO, high-WtHR, and EA (ORs 1.7, 1.6, and 1.9, respectively), and that hypertriglyceridemia was the other major independent factor associated with AO, high-WtHR, and EA (ORs 1.2, 1.2, and 1.4, respectively). Our data also supported the conclusions of some meta-analyses [40,41], which showed that AO and WtHR were better screening tools than BMI for detecting obesity-related cardiometabolic risk factors such as hypertension, DM, dyslipidemia, and CVD.

On the other hand, hyperuricaemia and renal and CVD outcomes form a difficult triad to unravel due to their relationships with myocardial infarction, heart failure, stroke, hypertension, DM, MetS, CKD, CVD, and all-cause death [42]. Hyperuricaemia promotes atherosclerosis and is associated with renal and cardiometabolic diseases [43,44]. However, it is a disorder that has not been included among the factors underlying CKM syndrome [23]. The results of our recent SIMETAP-HU study [45] showed that the increased WtHR was an independent factor associated with serum uric acid ≥ 7.0 mg/dL for both men and women in the overall adult population (OR 1.3), and that the AO was an independent factor associated with these hyperuricaemia levels in the male population (OR 1.7), and with serum uric acid ≥ 6.0 mg/dL in the female population (OR 2.3). Results from the present study showed that hyperuricaemia was an independent factor associated with AO (OR 1.2), high-WtHR (OR 1.5), and EA (OR 1.7); therefore, it is a metabolic variable that warrants further studies to investigate whether it can be included as another factor to be assessed among those subjects underlying stages 1 and 2 of CKM syndrome [23].

In addition to metabolic risk factors, stage 2 of CKM syndrome also includes individuals at moderate- to high-risk CKD [23]. The association of this risk was stronger with EA (OR 5.3) than with AO (OR 2.3) and with high-WtHR (OR 2.6). It is well known that obesity is a risk factor for the onset and progression of CKD [28], although the body fat indices are better predictors of CKD than BMI [46,47,48]. The Malmö Diet and Cancer Study (MDCS) cohort from the city of Malmö (Sweden) [49] showed that all anthropometric measures of obesity were associated with the risk of developing CKD in both men and women. However, body fat percentage was only associated with an increased risk of CKD in women (HR 2.0). Results of our SIMETAP-CKD study [50] showed than AO, EA, and increased WtHR were associated with CKD, but only increased WtHR was an independent factor associated with CKD (OR 1.6). The present study data showed that CKD was an independent factor associated with both high-WtHR and EA. However, although CKD was associated with AO, it did not reach statistical significance as an independent factor. This difference in AO as a predictor of risk CKD between the MDCS and our study might be due to the age difference between the Swedish population (45–73 years at baseline) and the overall adult population in our study, the lower obesity prevalence in Sweden (20.6%) than in Spain (23.8%) [2], and mainly to the fact that WCs were lower in the MDCS population than in our study (93.5 cm in men and 77.8 cm in women, vs. 98.0 cm and 89.7 cm, respectively). 

FLI values ≤ 30 can be used to rule out steatotic liver disease (SLD), and FLI values ≥ 60 can be used to rule in SLD [28]. We found a very close association of FLI ≥ 60 with AO, high-WtHR, and EA (Table 3 and Table S3, Supplementary Materials), which may support VAT excess, but FLI is a biased parameter in analyses involving these variables because it includes parameters such as WC and BMI. Nevertheless, excess VAT, regardless of BMI, has been related to insulin resistance and atherogenic factors such as hypertriglyceridemia and increased apolipoprotein B levels [5,51]. The correlation between epicardial adipose tissue density, the occurrence of CVD and MetS is widely recognized [5,52,53]. In visceral obesity, excess VAT is characterized by the hormone leptin hypersecretion and infiltration of activated macrophages, leading to the release of inflammatory cytokines (e.g., interleukin 6, tumour necrosis factor-α), which induce insulin resistance, endothelial dysfunction, hypercoagulability, and systemic inflammation, thereby facilitating the atherosclerotic process [5,52]. Our results showed that coronary heart disease, stroke, peripheral arterial disease, heart failure, and atrial fibrillation were associated with AO, high-WtHR, and EA, although none of them were independent factors associated with these adiposity-related variables.

A logical consequence of the accumulation of CKM risk factors was that about two-thirds of the population with AO, high-WtHR, or EA were at high or very high cardiovascular risk, which requires emphasis on the assessment of these adiposity-related variables and comprehensive action on all related diseases and clinical conditions mentioned above.

### 4.3. Strengths and Limitations

The main limitations of our study included the inability to preclude definitive conclusions about causal relationships or estimate incidence rates due to its observational design, variability between interviewers, and the heterogeneity of laboratory equipment and measurement precision. There may be some concern about bias in the results regarding WC measurement. We used the recommendations of the Spanish Society for the Study of Obesity (SEEDO, according to its acronym in Spanish) [54] in accordance with the National Cholesterol Education Program Third Adult Treatment Panel [55], which recommended measuring WC above the upper edge of the iliac crest in a horizontal plane. We chose this measure because it was the most well known among Spanish physicians, thus achieving greater homogeneity in the measurements. We agree that deviations may arise between measuring at this level or at the midpoint between the lowest ribs and the iliac crest, or at the narrowest waist, or at the level of the navel, although a systematic review of 120 studies with 236 samples concluded that the most common WC measurement protocols had no substantial influence on the association between WC, all-cause mortality and CVD, and DM and CVD [56].

On the other hand, there is a wide diversity of methods, models, and indices to estimate body fat and EA [7]. Techniques to assess adiposity, such as body composition analysis, were not used because bioimpedance devices that could assess body composition and VAT in individuals without body fluid and electrolyte abnormalities were not available. These records require morning measurements along with prior preparations such as fasting, not drinking stimulant beverages, not performing intense exercise during the previous 12 h, not having showered recently, and avoiding the menstruation period. We also did not analyze other indices to assess body composition, such as ABSI [8], based on WC, weight and height; waist-to-hip ratio, based on WC and hip circumference; the Conicity Index [9], based on WC, BMI, and height; the ARI 10], based on BMI and ABSI; the BRI [11], based on WC and height; the BAI [12], based on hip circumference and height; and the VAI [13], based on WC, BMI, HDL-C levels, and triglyceride levels. In our study, not all subjects had their HDL-C levels recorded, which was necessary to measure VAI. Furthermore, age is a factor that is closely related to the increase in prevalence of the three variables assessed in this study, and yet none of the aforementioned indices includes age in their definition algorithms. On the contrary, the CUN-BAE [14] used in our study does include it.

Key strengths include a large sample of people aged from 18 to 102 years recruited using a random, population-based method. Assessing the epidemiological magnitude of adiposity-related variables is essential to better plan prevention policies aimed to optimize available health resources. The results reported herein are biologically plausible and consistent with the available scientific information; they also update the adjusted prevalence rates of adiposity-related variables in the overall population and may help to better understand their clinical characteristics and their association with CKM syndrome factors.

## 5. Conclusions

AO, high-WtHR, and EA are good indices for assessing adiposity, but are less used than BMI in clinical practice. However, restricting the diagnosis of obesity to BMI measurement underestimates the assessment of excessive body fat accumulation. Two-thirds of the adult population have EA, one-third have AO, and one-third have high-WtHR. Our results support that diagnostic criteria for both MetS and stages 1 and 2 of CKM syndrome, such as hypertension, hypertriglyceridemia, low HDL-c, and adiposity dysfunction reflected as prediabetes or diabetes, are independent factors associated with AO, high-WtHR, and EA, highlighting hypertension. In addition, physical inactivity and hyperuricemia, factors not already included in CKM syndrome, also show an independent association with these adiposity-related variables.

## Figures and Tables

**Table 1:** Anthropometric parameters of the study population.

	Overall Population		Male Population		Female Population		*p*-Value *
	Mean (SD)	Median (IQR)	Mean (SD)	Median (IQR)	Mean (SD)	Median (IQR)	
Weight cm	74.2 (15.6)	73.0 (63.0–83.9)	81.8 (14.5)	80.0 (72.0–90.0)	68.3 (13.8)	66.0 (58.5–76.0)	<0.001
Height cm	164.2 (9.8)	164.0 (157.0–171.0)	171.0 (7.9)	171.0 (166.0–176.0)	158.8 (7.4)	159.0 (154.0–164.0)	<0.001
BMI kg/m^2^	27.5 (5.1)	27.0 (23.9–30.5)	27.9 (4.5)	27.5 (24.8–30.5)	27.2 (5.6)	26.4 (23.1–30.5)	0.158
WC cm	93.4 (14.1)	93.0 (83.0–102.0)	98.0 (12.6)	98.0 (90.0–105.0)	89.7 (14.1)	87.5 (79.0–99.0)	<0.001
WtHR	0.57 (0.09)	0.56 (0.51–0.63)	0.57 (0.08)	0.57 (0.52–0.62)	0.57 (0.10)	0.56 (0.49–0.63)	1
CUN-BAE%	34.7 (8.7)	34.2 (28.5–41.1)	28.9 (6.3)	29.2 (25.1–33.0)	39.3 (7.5)	39.8 (34.1–44.9)	<0.001

* *p*-value of difference in means between male and female populations. BMI: body mass index; CUN-BAE: according to its acronym in Spanish, *Clínica Universitaria de Navarra*—Body Adiposity Estimator; IQR: interquartile range; SD: standard deviation; WC: waist circumference; WtHR: waist-to-height ratio.

**Table 2:** Prevalence rates of abdominal obesity, high-WtHR, and excess adiposity.

	Crude Prevalence Rates				Adjusted Prevalence Rates		
	Overall % (95% CI)	Male % (95% CI)	Female % (95% CI)	*p*	Overall (%)	Male (%)	Female (%)
AO	44.4 (43.2–45.6)	37.9 (36.2–39.7)	49.4 (47.8–51.0)	<0.001	39.6	33.6	44.9
High-WtHR	35.8 (34.7–37.0)	36.8 (35.1–38.6)	35.1 (33.5–36.6)	0.144	30.6	31.1	30.6
EA	73.3 (72.3–74.4)	75.4 (73.8–76.9)	71.7 (70.3–73.2)	0.001	65.6	66.7	65.3

AO: abdominal obesity; CI: confidence interval; EA: excess adiposity; *p*: *p*-value of difference in percentages; WtHR: waist-to-height ratio.

**Table 3:** Clinical characteristics of populations with and without AO, high-WtHR, and EA.

	With AO No. 2922	Without AO No. 3666	*p*	Cohen’s *d* (95% CI)	With High-WtHR No. 2361	Without High-WtHR No. 4227	*p*	Cohen’s *d* (95% CI)	With EA No. 4832	Without EA No. 1756	*p*	Cohen’s *d* (95% CI)
	Mean (SD)	Mean (SD)			Mean (SD)	Mean (SD)			Mean (SD)	Mean (SD)		
Age yr	60.7 (15.8)	50.7 (17.6)	<0.001	0.6 (0.6; 0.6)	63.6 (15.1)	50.4 (17.0)	<0.001	0.8 (0.8; 0.9)	60.5 (15.8)	40.3 (13.0)	<0.001	1.3 (1.3; 1.4)
BMI kg/m^2^	31.3 (4.6)	24.5 (3.3)	<0.001	1.7 (1.7; 1.8)	32.1 (4.5)	24.9 (3.4)	<0.001	1.9 (1.8; 1.9)	29.5 (4.5)	22.1 (2.2)	<0.001	1.8 (1.8; 1.9)
WC cm	104.7 (10.3)	84.3 (9.4)	<0.001	2.1 (2.0; 2.1)	107.1 (9.5)	85.7 (9.7)	<0.001	2.2 (2.2; 2.3)	98.1 (12.5)	80.3 (9.1)	<0.001	1.5 (1.5; 1.6)
WtHR	0.64 (0.06)	0.51 (0.05)	<0.001	2.4 (2.3; 2.4)	0.66 (0.06)	0.52 (0.05)	<0.001	2.6 (2.6; 2.7)	0.60 (0.08)	0.48 (0.05)	<0.001	1.6 (1.6; 1.7)
CUN-BAE adiposity	40.6 (7.0)	30.0 (6.8)	<0.001	1.5 (1.5; 1.6)	41.0 (7.3)	31.3 (7.3)	<0.001	1.3 (1.3; 1.4)	37.8 (7.4)	26.2 (5.8)	<0.001	1.6 (1.6; 1.7)
SBP mmHg	126.5 (14.9)	118.3 (14.9)	<0.001	0.6 (0.5; 0.6)	127.9 (14.7)	118.6 (14.9)	<0.001	0.6 (0.6; 0.7)	125.2 (14.7)	113.0 (14.0)	<0.001	0.8 (0.8; 0.9)
DBP mmHg	75.7 (9.4)	71.4 (9.7)	<0.001	0.5 (0.4; 0.5)	76.0 (9.2)	71.8 (9.7)	<0.001	0.4 (0.4; 0.5)	75.0 (9.3)	68.7 (9.5)	<0.001	0.7 (0.6; 0.7)
FPG mg/dL ^a^	101.6 (28.8)	91.6 (22.5)	<0.001	0.4 (0.3; 0.4)	104.2 (30.5)	91.4 (21.7)	<0.001	0.5 (0.5; 0.6)	99.4 (27.1)	86.7 (19.8)	<0.001	0.5 (0.5; 0.6)
HbA_1c_ % ^b,^*	5.84 (0.94)	5.46 (0.82)	<0.001	0.4 (0.4; 0.5)	5.94 (1.01)	5.45 (0.76)	<0.001	0.6 (0.5; 0.6)	5.77 (0.92)	5.24 (0.68)	<0.001	0.6 (0.6; 0.7)
TC mg/dL ^c^	194.7 (39.6)	191.3 (39.0)	0.001	0.1 (0.0; 0.1)	193.3 (38.6)	192.5 (39.8)	0.400	0.0 (0.0; 0.1)	195.0 (39.7)	186.7 (37.7)	<0.001	0.2 (0.2; 0.3)
HDL-C mg/dL ^c^	52.6 (13.9)	56.6 (15.1)	<0.001	−0.3 (−0.6; −0.2)	51.8 (13.6)	56.5 (15.0)	<0.001	−0.3 (−0.4; −0.3)	53.4 (14.3)	58.7 (15.1)	<0.001	−0.4 (−0.4; −0.3)
LDL-C mg/dL ^c,^*	115.6 (35.0)	113.0 (34.1)	0.003	0.1 (0.0; 0.1)	114.4 (34.0)	114.0 (34.8)	0.664	0.0 (0.0; 0.1)	116.1 (34.8)	108.7 (33.1)	<0.001	0.2 (0.2; 0.3)
RC mg/dL ^c,^*	26.0 (12.8)	20.9 (11.2)	<0.001	0.4 (0.4; 0.5)	26.6 (12.8)	21.2 (11.4)	<0.001	0.4 (0.4; 0.5)	24.7 (12.4)	18.8 (10.5)	<0.001	0.5 (0.4; 0.6)
Non-HDL-C mg/dL ^c^	142.1 (38.3)	134.6 (38.2)	<0.001	0.2 (0.2; 0.2)	141.5 (37.2)	136.0 (39.0)	<0.001	0.1 (0.1; 0.2)	141.5 (38.2)	128.0 (37.3)	<0.001	0.4 (0.3; 0.4)
TG mg/dL ^d^	134.0 (77.2)	109.8 (86.2)	<0.001	0.3 (0.3; 0.3)	137.0 (76.5)	111.3 (85.4)	<0.001	0.3 (0.3; 0.4)	128.7 (80.4)	98.1 (86.5)	<0.001	0.4 (0.3; 0.4)
TG/HDL-C	2.88 (2.31)	2.24 (2.70)	<0.001	0.2 (0.2; 0.3)	2.98 (2.32)	2.27 (2.64)	<0.001	0.3 (0.2; 0.4)	2.75 (2.39)	1.93 (2.87)	<0.001	0.3 (0.3; 0.4)
SUA mg/dL ^e,^*	5.25 (1.49)	4.74 (1.44)	<0.001	0.4 (0.3; 0.4)	5.41 (1.52)	4.72 (1.40)	<0.001	0.5 (0.4; 0.5)	5.18 (1.49)	4.39 (1.28)	<0.001	0.6 (0.5; 0.6)
AST U/L *	23.2 (18.0)	23.0 (55.8)	0.832	0.0 (0.0; 0.1)	23.3 (19.0)	22.9 (52.0)	0.743	0.0 (0.0; 0.1)	23.3 (36.9)	22.5 (56.9)	0.551	0.0 (0.0; 0.1)
ALT U/L *	26.4 (18.8)	23.7 (15.2)	<0.001	0.2 (0.1; 0.2)	26.5 (19.3)	24.0 (15.4)	<0.001	0.2 (0.1; 0.2)	25.7 (17.2)	22.7 (15.9)	<0.001	0.2 (0.1; 0.2)
GGT U/L *	38.8 (65.5)	29.2 (34.5)	<0.001	0.2 (0.1; 0.2)	39.2 (51.4)	30.3 (50.1)	<0.001	0.2 (0.1; 0.2)	36.5 (55.7)	25.2 (32.3)	<0.001	0.2 (0.2; 0.3)
FLI 0–100	67.9 (23.4)	27.2 (22.4)	<0.001	1.8 (1.7; 1.8)	73.5 (19.9)	29.5 (23.2)	<0.001	2.0 (1.9; 2.0)	55.8 (27.5)	15.9 (15.6)	<0.001	1.6 (1.6; 1.7)
Creatinine mg/dL ^f^	0.84 (0.28)	0.84 (0.30)	0.837	0.0 (−0.1; 0.1)	0.87 (0.31)	0.83 (0.28)	<0.001	0.1 (0.1; 0.2)	0.85 (0.29)	0.81 (0.30)	<0.001	0.1 (0.1; 0.2)
eGFR mL/min/1.73 m^2^	86.0 (20.3)	94.2 (20.0)	<0.001	−0.4 (−0.5; −0.4)	83.8 (20.5)	94.3 (19.6)	<0.001	−0.5 (−0.6; −0.5)	86.3 (19.9)	102.3 (17.4)	<0.001	−0.8 (−0.9; −0.8)
uACR mg/g ^g^	19.3 (69.8)	14.1 (51.6)	<0.001	0.1 (0.0; 0.1)	22.7 (78.9)	12.9 (46.8)	<0.001	0.2 (0.1; 0.2)	19.1 (69.2)	9.1 (21.2)	<0.001	0.2 (0.1; 0.2)

CI: confidence interval; Cohen’s *d*; effect size of standardized mean difference according to the proximity to the following absolute *d*-values: 0.2 small, 0.5 medium, 0.8 large; *p*: *p*-value of the difference in means; SD: standard deviation. AO: abdominal obesity; WtHR: waist-to-height ratio; EA: excess adiposity. ALT: alanine aminotransferase (* No. with vs. without AO: 2845 vs. 3577; No. with vs. without high-WtHR: 2294 vs. 4128; No. with vs. without EA: 4703 vs. 1719); AST: aspartate aminotransferase (* No. with vs. without AO: 2165 vs. 2656; No. with vs. without high-WtHR: 1735 vs. 3086; No. with vs. without EA: 3533 vs. 1288); BMI: body mass index; CUN-BAE-adiposity: according to its acronym in Spanish, *Clínica Universitaria de Navarra*—Body Adiposity Estimator; DBP: diastolic blood pressure; eGFR: estimated glomerular filtration rate; FGP: fasting plasma glucose; FLI: fatty liver index (* No. with vs. without AO: 2690 vs. 3418; No. with vs. without high-WtHR: 2613 vs. 3945; No. with vs. without EA: 4470 vs. 1638); GGT: gamma-glutamyl transferase (* No. with vs. without AO: 2690 vs. 3418; No. with vs. without high-WtHR: 2163 vs. 3945; No. with vs. without EA: 4470 vs. 1638); HbA_1c_: glycated hemoglobin A_1c_ (* No. with vs. without AO: 2439 vs. 2794; No. with vs. without high-WtHR: 2001 vs. 3232; No. with vs. without EA: 3946 vs. 1287); HDL-C: high-density lipoprotein cholesterol; LDL-C: low-density lipoprotein cholesterol (* No. with vs. without AO: 2892 vs. 3634; No. with vs. without high-WtHR: 2339 vs. 4187; No. with vs. without EA: 4779 vs. 1747); RC: residual cholesterol (* No. with vs. without AO: 2892 vs. 3634; No. with vs. without high-WtHR: 2339 vs. 4187; No. with vs. without EA: 4779 vs. 1747); SBP: systolic blood pressure; SUA: serum uric acid (* No. with vs. without AO: 2878 vs. 3611; No. with vs. without high-WtHR: 2319 vs. 4170; No. with vs. without EA: 4755 vs. 1734); TC: total cholesterol; TG: triglycerides; uACR: urine albumin/creatinine ratio; WC: waist circumference. The definitions of the variables are shown in Table S1 (Supplementary Materials). ^a^ To convert from mg/dL to mmol/L, multiply by 0.05556. ^b^ To convert from % (DCCT) to mmol/mol (IFCC), subtract 2.15 and multiply by 10.929. ^c^ To convert from mg/dL to mmol/L, multiply by 0.02586. ^d^ To convert from mg/dL to mmol/L, multiply by 0.01129. ^e^ To convert from mg/dL to mmol/L, multiply by 0.05948. ^f^ To convert from mg/dL to mmol/L, multiply by 0.08842. ^g^ To convert from mg/g to mg/mmol, multiply by 0.01131.

## Data Availability

The original contributions presented in the study are included in the article/Supplementary Materials, further inquiries can be directed to the corresponding author.

## Supplementary Materials

The following supporting information can be downloaded at: https://www.mdpi.com/article/10.3390/nu16223948/s1, Figure S1. Flowchart of the sampling and selection of study subjects; Table S1. Definitions and criteria of clinical conditions and variables; Table S2. Anthropometric parameters of populations with AO, high-WtHR, and EA; Figure S2. (A) Relationship between age and WC in people with AO in men and women; (B) relationship between age and WtHR in people with high-WtHR in men and women; (C) relationship between age and CUN-BAE in people with EA in men and women; Table S3. Correlations among anthropometric parameters; Table S4. Comorbidities and medical conditions in populations with and without AO, high-WtHR, and EA; Table S5. Multivariate analysis of factors and medical conditions for AO, high-WtHR, and EA [4,14,22,26,27,28,54,55,57,58,59,60,61,62,63,64,65,66].

## Abbreviations

ABSI	a body shape index
AO	abdominal obesity
ARI	anthropometric risk index
BAI	body adiposity index
BMI	body mass index
BRI	body roundness index
CKD	chronic kidney disease
CKM	cardiovascular–kidney–metabolic
CUN-BAE	*Clínica Universitaria de Navarra*—Body Adiposity Estimator
CVD	cardiovascular disease
DM	diabetes mellitus
EA	excess adiposity
FLI	fatty liver index
HDL-c	high-density lipoprotein cholesterol
MetS	metabolic syndrome
SLD	steatotic liver disease
T2DM	type2 diabetes mellitus
VAI	visceral adiposity index
VAT	visceral adipose tissue
WtHR	waist-to-height ratio
