# Supplementary material for: Prevalence Rates of Abdominal Obesity, High Waist-to-Height Ratio and Excess Adiposity, and Their Associated Cardio-Kidney-Metabolic Factors: SIMETAP-AO Study
Source: Nutrients. 2024 Nov 19;16(22):3948. doi: 10.3390/nu16223948 (PMC11597375; doi:10.3390/nu16223948)
Supplement: Supplementary file 1 [file nutrients-16-03948-s001.zip › nutrients-3250578-supplementary.pdf]

Prevalence rates of abdominal obesity, high waist-to-height ratio and excess adiposity, and their associated cardio-kidney-metabolic factors. SIMETAP-AO Study.

SUPPLEMENTARY MATERIALS

Figure S1. Flowchart for sampling and selection of study subjects

Table S1. Definitions and criteria of clinical conditions and variables

Table S2. Anthropometric parameters of populations with AO, high-WtHR and EA

Figure S2. A) Relationship between age and WC in people with AO in men and women B) Relationship between age and WtHR in people with high-WtHR in men and women C) Relationship between age and CUN-BAE in people with EA in men and women

Table S3. Correlations among anthropometric parameters

Table S4. Comorbidities and medical conditions in populations with and without AO, high-WtHR, and EA

Table S5. Multivariate analysis of factors and medical conditions for AO, high-WtHR, and EA

Figure S1. Flowchart for sampling and selection of study subjects

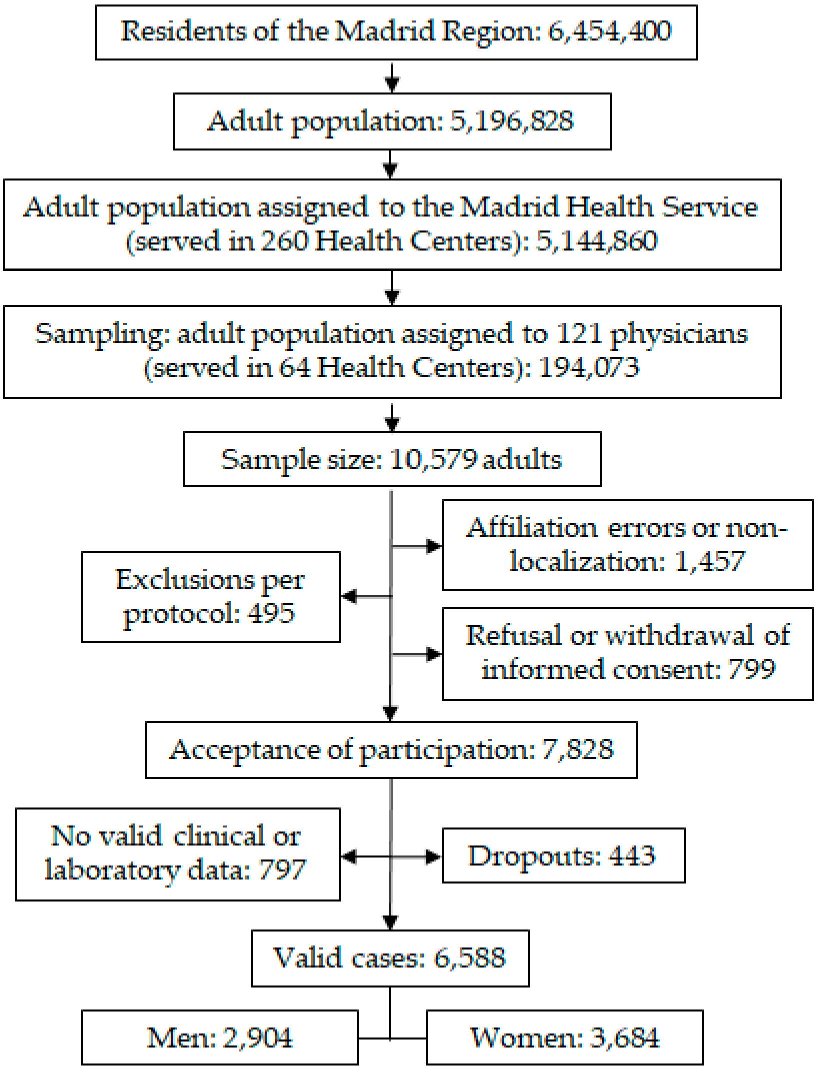

Table S1. Definitions and criteria of clinical conditions and variables

**Morbidities, variables or clinical conditions**	**Definitions and criteria**
Current smoking	Any amount of tobacco use in the previous year.
Alcoholism	> 21 standard drink units (SDU) of alcohol consumption per week (male), > 14 SDU per week (female).<br>1 SDU is equivalent to 10 g of alcohol.
Physical inactivity (1)	Moderate-intensity physical activity (e.g., brisk walking) < 150 minutes a week, or vigorous-intensity physical activity (e.g., jogging) < 75 minutes a week (or less of equivalent combination of both), or muscle strengthening exercises < 2 days a week.
Body mass index (BMI)	BMI was calculated as weight in kilograms divided by the square of height in meters.
Overweight (2)	BMI 25.0–29.9 kg/m^2 (International Classification of Diseases, 10th Revision, Clinical Modification [ICD-10-CM]: E66.3; International Classification of Primary Care, 2nd edition [ICPC-2]: T83)*
Obesity (2)	BMI ≥ 30 kg/m^2 (ICD-10-CM: E66.9; ICPC-2: T82)*
Waist circumference (WC) (3,4)	WC determined with the subject standing using a flexible tape measure adjusted without compressing the skin, after the participant exhaled a normal breath, locating the upper edge of the iliac crests and above that point surrounding the waist parallel to the floor.
Abdominal (or central) obesity (AO) (5)	WC ≥ 102 cm in men or ≥ 88 cm in women
Waist-to-height ratio (WtHR)	WtHR is calculated as WC measurement divided by height measurement, both in centimetres.
High-WtHR (6)	WtHR ≥ 0.60 for both male and female populations.
CUN-BAE adiposity (7)	CUN-BAE (according to its acronym in Spanish, *Clínica Universitaria de Navarra* - Body Adiposity Estimator) body fat index:<br>• Male: $-44.988 + (0.503 \times age) + (3.172 \times BMI) - (0.026 \times BMI^2) - (0.02 \times BMI \times age) + (0.00021 \times BMI^2 \times age)$<br>• Female: $-44.988 + (0.503 \times age) + 10.689 + (3.172 \times BMI) - (0.026 \times BMI^2) + (0.181 \times BMI) - (0.02 \times BMI \times age) - (0.005 \times BMI^2) + (0.00021 \times BMI^2 \times age)$
CUN-BAE excess adiposity (EA) (7)	CUN-BAE body fat index > 25% for men or > 35% for women

Arterial hypertension (HTN) (8)	Systolic blood pressure (SBP) ≥ 140 mmHg and/or diastolic blood pressure (DBP) ≥ 90 mmHg, using the average of two or more readings obtained on two or more occasions, or being on blood pressure-lowering drug therapy (BPLT) (ICD-10-CM: I10, I15; ICPC-2: K86, K87)*
Pulse pressure	SBP – DBP (mmHg)
Diabetes mellitus (DM) (9)	According to the American Diabetes Association (ADA) criteria: fasting plasma glucose (FPG) ≥ 126 mg/dL (7.0 mmol/L) or glycated haemoglobin A_{1c} (HbA_{1c}) ≥ 6.5 % (in International Federation of Clinical Chemistry and Laboratory Medicine [IFCC] units) (≥ 48 mmol/mol) or plasma glucose ≥ 200 mg/dL (11.1 mmol/L) at any time or with oral glucose tolerance test (ICD-10-CM: E10, E11; ICPC-2: T89, T90)*<br>To convert from mg/dL to mmol/L, multiply by 0.05556<br>To convert from % (DCCT) to mmol/mol (IFCC), subtract 2.15 and multiply by 10.929
Prediabetes (9)	According to the ADA criteria: FPG between 100 and 125 mg/dL or HbA_{1c} between 5.7% and 6.4% (ICD-10-CM: R73.09; ICPC-2: A91)*<br>To convert from mg/dL to mmol/L, multiply by 0.05556<br>To convert from % (DCCT) to mmol/mol (IFCC), subtract 2.15 and multiply by 10.929
Estimated average glucose (eAG)	28.7 x HbA_{1c} – 46.7 (mg/dL)<br>To convert from mg/dL to mmol/L, multiply by 0.05556
Hypercholesterolaemia	Fasting plasma total cholesterol (TC) concentration ≥ 200 mg/dL (≥ 5.17 mmol/L) (ICD-10-CM: E78; ICPC-2: T93)* or being on lipid-lowering drug therapy (LLT) to achieve cholesterol targets.<br>To convert from mg/dL to mmol/L, multiply by 0.02586
Hypertriglyceridaemia (HTG)	Fasting plasma triglycerides (TG) concentration ≥ 150 mg/dL (≥ 1.69 mmol/L) (ICD-10-CM: E78; ICPC-2: T93)* or being on triglycerides lowering specific drug therapy.<br>To convert from mg/dL to mmol/L, multiply by 0.01129
Low high-density lipoprotein cholesterol (HDL-C)	HDL-C < 40 mg/dL (< 1.03 mmol/L) (for men)<br>HDL-C < 50 mg/dL (< 1.29 mmol/L) (for women)<br>To convert from mg/dL to mmol/L, multiply by 0.02586
Atherogenic dyslipidaemia	HTG and low HDL-C
Non-high-density lipoprotein cholesterol (Non-HDL-C)	TC – HDL-C
Low-density lipoprotein cholesterol (LDL-C)	TC – HDL-C – (TG/5) mg/dL (not valid for patients with TG > 400 mg/dL)<br>TC – HDL-C – (TG/2.2) mmol/L (not valid for patients with TG > 4.51 mmol/L)<br>TC, HDL-C: To convert from mg/dL to mmol/L, multiply by 0.02586<br>TG: To convert from mg/dL to mmol/L, multiply by 0.01129

Residual cholesterol (RC)	Very low-density lipoproteins cholesterol (VLDL-C) and remnants.<br>RC= TC – HDL-C – LDL-C
Atherogenic index of plasma (AIP)	log TG/HDL
Triglyceride-glucose (TyG) index	Ln (TG x FPG/2)
Metabolic syndrome (MetS) (5)	According to Harmonized Consensus of International Diabetes Federation task force on Epidemiology and Prevention, National Heart, Lung, and Blood Institute, American Heart Association, World Heart Federation, International Atherosclerosis Society, and International Association for the Study of Obesity (3).<br>At least, three of following factors for the European population:<br>• Increased WC (≥ 102 cm for men; ≥ 88 cm for women)<br>• FPG ≥ 100 mg/dL (≥ 5.6 mmol/L)<br>• TG ≥ 150 mg/dL (≥ 1.7 mmol/L)<br>• HDL-C < 40 mg/dL (< 1.03 mmol/L) (males); < 50 mg/dL (< 1.29 mmol/L) (females)<br>• SBP ≥ 130 mmHg or DBP ≥ 85 mmHg or antihypertensive treatment
Fatty liver index (FLI) (10)	FLI = $(e^{0.953*\log_e(TG) + 0.139 \times BMI + 0.718 \times \log_e(GGT) + 0.053 \times \text{waist circumference} - 15.745}) / (1 + e^{0.953*\log_e(TG) + 0.139 \times BMI + 0.718 \times \log_e(GGT) + 0.053 \times \text{waist circumference} - 15.745}) \times 100$<br>$\log_e$ = natural logarithm; GGT = gamma-glutamyl-transferase<br>A value FLI between 0 and 30 can be used to rule out steatotic liver disease (SLD) (sensitivity: 87%; negative likelihood ratio: 0.2)<br>A value FLI between 60 and 100 can be used to rule in SLD (specificity 86%; positive likelihood ratio: 4.3).
Hyperuricaemia (HU) (11)	Serum uric acid (SUA) levels ≥ 7.0 mg/dL (416 μmol/L) for both adult male and female populations, or being on urate-lowering therapy (ULT) (ICD-10-CM: E79; ICPC-2: T92)*<br>To convert from mg/dL to mmol/L, multiply by 0.05948
Coronary heart disease (CHD)	Ischemic heart disease, acute myocardial infarction, acute coronary syndrome, coronary revascularization (ICD-10-CM: I20-I25; ICPC-2: K74, K75, K76)*
Cerebrovascular disease (stroke)	Cerebral ischemia, intracranial haemorrhage, transient ischemic attack (ICD-10-CM: I60-I66, I66, I67; ICPC-2: K89, K90K K91)*
Peripheral arterial disease (PAD)	Intermittent claudication, ankle-brachial index ≤ 0.9 (ICD-10-CM: I70.2, I73.9; ICPC-2: K92)*
Atherosclerotic cardiovascular disease (ASCVD)	ASCVD include CHD, stroke, or PAD (ICD-10-CM: I70)*

Heart failure (HF) (12)	Record of HF diagnosis (ICD-10-CM: I50; ICPC-2: K77)* in the primary care electronic health records, without differentiating by phenotype based on measurement of left ventricular ejection fraction or based on severity of symptoms and physical activity.
Atrial fibrillation (AF) (13,14)	Record of AF diagnosis (ICD-10-CM: I48; ICPC-2: K78)* in the primary care electronic health records, without differentiating by phenotypes based on paroxysmal, persistent, long-standing persistent, or permanent AF or atrial flutter.
Estimated glomerular filtration rate (eGFR) (15)	According to Chronic Kidney Disease Epidemiology Collaboration (CKD-EPI) equations:<br>Women with creatinine ≤ 0.7 mg/dL= 144 x $(\text{creatinine})^{-0.329}$ x $(0.993)^{\text{age}}$ mL/min/1.73 m^2 of the body surface<br>Women with creatinine > 0.7 mg/dL= 144 x $(\text{creatinine})^{-1.209}$ x $(0.993)^{\text{age}}$ mL/min/1.73 m^2 of the body surface<br>Men with creatinine ≤ 0.9 mg/dL= 141 x $(\text{creatinine})^{-0.411}$ x $(0.993)^{\text{age}}$ mL/min/1.73 m^2 of the body surface<br>Men with creatinine > 0.9 mg/dL= 141 x $(\text{creatinine})^{-1.209}$ x $(0.993)^{\text{age}}$ mL/min/1.73 m^2 of the body surface
Low eGFR (16)	eGFR < 60 mL/min/1.73 m^2 according to CKD-EPI [12]<br>Low eGFR does not include the following categories:<br>• G1: ≥ 90 mL/min/1.73 m^2<br>• G2: 60 to 89 mL/min/1.73 m^2<br>Low eGFR includes the following categories:<br>• G3a: 45 to 59 mL/min/1.73 m^2<br>• G3b: 30 to 44 mL/min/1.73 m^2<br>• G4: 15 to 29 mL/min/1.73 m^2<br>• G5: < 15 mL/min/1.73 m^2
Albuminuria (16)	Urine albumin-creatinine ratio (uACR) ≥ 30 mg/g (including proteinuria [uACR > 300 mg/g] (ICD-10-CM: R80; ICPC-2: U98)*<br>Albuminuria does not include the following category:<br>• A1: < 30 mg/g<br>Albuminuria includes the following categories:<br>• A2: 30 mg/g to 300 mg/g<br>• A3: > 300 mg/g<br>To convert from mg/g to mg/mmol, multiply by 0.01131
Chronic kidney disease (CKD) (16)	Low eGFR and/or albuminuria (ICD-10-CM: N18; ICPC-2: U99)*
Risk CKD categories (16)	Risk of CKD progression, acute kidney injury, kidney failure replacement therapy, all-cause mortality and cardiovascular events.<br>• Low risk: G1A1; G2A1<br>• Moderate risk: G1A2; G2A2; G3aA1<br>• High risk: G1A3; G2A3; G3aA2; G3bA1<br>• Very high risk: G3aA3; G3bA2; G3bA3; G4A1; G4A2; G4A3; G5A1; G5A2; G5A3

Cardiovascular risk (CVR) categories (17)	Ten-year fatal and non-fatal cardiovascular disease (CVD) risk for patients from low-risk European countries.<br>Low, moderate, high and very high CVR categories were estimated according to 2021 ESC Guidelines on cardiovascular disease prevention in clinical practice.
Cardiovascular-kidney-metabolic (CKM) syndrome (18)	Systemic disorder attributable to pathophysiological interactions among metabolic risk factors, CKD, and CVD, that includes both individuals at risk for CVD, CKD, and those with existing clinical CVD.<br>• Stage 0: BMI <25 kg/m^2, normal abdominal circumference (<88 in women and <102 cm in men) without criteria for the other stages.<br>• Stage 1: adiposity excess (CUN-BAE obesity), overweight, obesity, abdominal obesity, or prediabetes.<br>• Stage 2: HTN, metabolic risk factors (HTG, DM, MetS), moderate or high risk CKD.<br>• Stage 3: subclinical target organ damage, risk equivalents (high CVR or very high-risk CKD) among individuals with stages 1 or 2.<br>• Stage 4: clinical CVD including CHD, stroke, PAD, HF, and AF among individuals with stages 1 or 2 (stage 4a: without CKD; stage 4b: with CKD).<br>• Stages 3 or 4 are defined as advanced stages of CKM syndrome because they identify individuals at high risk for CVD.

* National Center for Health Statistics (NCHS). International Classification of Diseases, Tenth Revision, Clinical Modification (ICD-10-CM). [Accessed September 22, 2024]. Available from: https://www.cdc.gov/nchs/icd/icd-10-cm/index.html.

* World Health Organization. (2009). International Classification of Primary Care, 2nd edition -ICPC-2. [Accessed September 22,, 2024]. Available from: https://www.who.int/standards/classifications/other-classifications/international-classification-of-primary-care.

References

1. World Health Organization. WHO guidelines on physical activity and sedentary behaviour. Geneva: World Health Organization. **2020**. [Accessed September 22, 2024]. Available from: https://iris.who.int/handle/10665/336656.
2. WHO Consultation on Obesity (1999: Geneva, Switzerland) & World Health Organization). Obesity: preventing and managing the global epidemic: report of a WHO consultation. WHO technical report series; 894. **2000**. [Accessed September 22, 2024]. Available from: https://apps.who.int/iris/handle/10665/42330.
3. Salas-Salvadó J, Rubio MA, Barbany M, Moreno B, Grupo Colaborativo de la SEEDO. Consenso SEEDO 2007 para la evaluación del sobrepeso y la obesidad y el establecimiento de criterios de intervención terapéutica. Med Clin (Barc) **2007**; 128(5):184–96 [Accessed September 22, 2024]. Available from: https://doi.org/10.1016/S0025-7753(07)72531-9.
4. National Cholesterol Education Program (NCEP) Expert Panel on Detection, Evaluation, and Treatment of High Blood Cholesterol in Adults (Adult Treatment Panel III). Third Report of the National Cholesterol Education Program (NCEP) Expert Panel on Detection, Evaluation, and Treatment of High Blood Cholesterol in Adults (Adult Treatment Panel III) final report. Circulation. **2002**; 106:3143–421. https://doi.org/10.1161/01.cir.0000048067.86569.e1.
5. Alberti KGMM, Eckel RH, Grundy SM, Zimmet PZ, Cleeman JI, Donato KA, et al. Harmonizing the metabolic syndrome: A joint interim statement of the International Diabetes Federation task force on Epidemiology and Prevention; National Heart, Lung, and Blood Institute; American Heart Association; World Heart Federation; International Atherosclerosis Society; and International Association for the Study of Obesity. Circulation. **2009**; 120:1640–5, https://doi.org/10.1161/CIRCULATIONAHA.109.192644
6. National Institute for Health and Care Excellence (NICE). **2024**. Obesity: identification, assessment and management. Clinical guideline [CG189]. [Accessed September 22, 2024]. Available from: www.nice.org.uk/guidance/cg189.

7. Gómez-Ambrosi J, Silva C, Catalán V, Rodríguez A, Galofré JC, Escalada J, et al. Clinical usefulness of a new equation for estimating body fat. Diabetes Care. **2012**; 35:383–8, https://doi.org//10.2337/dc11-1334.
8. McEvoy JW, McCarthy CP, Bruno RM, Brouwers S, Canavan MD, Ceconi C, et al., ESC Scientific Document Group. 2024 ESC Guidelines for the management of elevated blood pressure and hypertension: Developed by the task force on the management of elevated blood pressure and hypertension of the European Society of Cardiology (ESC) and endorsed by the European Society of Endocrinology (ESE) and the European Stroke Organisation (ESO). Eur Heart J. **2024**; 00:1–107, ehae178. https://doi.org/10.1093/eurheartj/ehae178.
9. American Diabetes Association Professional Practice Committee. 2. Diagnosis and classification of diabetes: Standards of Care in Diabetes—2024. Diabetes Care. **2024**; 47(Suppl. 1):S20–S42, https://doi.org/10.2337/dc24-S002
10. Bedogni G, Bellentani S, Miglioli L, Masutti F, Passalacqua M, Castiglione A, et al. The Fatty Liver Index: a simple and accurate predictor of hepatic steatosis in the general population. BMC Gastroenterol. **2006**; 6:33. https://doi.org/10.1186/1471-230X-6-33.
11. Khanna D, Fitzgerald JD, Khanna PP, Bae S, Singh MK, Neogi T, et al. 2012 American College of Rheumatology Guidelines for Management of Gout Part I: systematic nonpharmacologic and pharmacologic therapeutic approaches to hyperuricemia. Arthritis Care Res. **2012**; 64,1431–46, https://doi.org/10.1002/acr.21772.
12. Abraham WT, Psotka MA, Fiuzat M, Filippatos G, Lindenfeld J, Mehran R, et al. Standardized definitions for evaluation of heart failure therapies: Scientific Expert Panel from the Heart Failure Collaboratory and Academic Research Consortium. JACC Heart Fail. **2020**; 8(12):961-972. https://doi.org/10.1016/j.jchf.2020.10.002.
13. Hindricks G, Potpara T, Dagres N, Arbelo E, Bax JJ, Blomström-Lundqvist C, et al.; ESC Scientific Document Group. 2020 ESC Guidelines for the diagnosis and management of atrial fibrillation developed in collaboration with the European Association for Cardio-Thoracic Surgery (EACTS): The Task Force for the diagnosis and management of atrial fibrillation of the European Society of Cardiology (ESC). Developed with the special contribution of the European Heart Rhythm Association (EHRA) of the ESC. Eur. Heart. J. **2021**; 42:373–498. https://doi.org/10.1093/eurheartj/ehaa612.
14. Joglar JA, Chung MK, Armbruster AL, Benjamin EJ, Chyou JY, Cronin EM, et al. 2023 ACC/AHA/ACCP/HRS Guideline for the diagnosis and management of atrial fibrillation: A report of the American College of Cardiology/American Heart Association Joint Committee on Clinical Practice Guidelines. Circulation. **2024**; 149(1):e1-e156. https://doi.org/10.1161/CIR.0000000000001193.
15. Levey AS, Stevens LA, Schmid CH, Zhang YL, Castro AF 3rd, Feldman HI, et al., CKD-EPI (Chronic Kidney Disease Epidemiology Collaboration). A new equation to estimate glomerular filtration rate. Ann Intern Med. **2009**; 150:604–12, https://doi.org/10.7326/0003-4819-150-9-200905050-00006.
16. Kidney Disease: Improving Global Outcomes (KDIGO) CKD Work Group. KDIGO 2024 Clinical Practice Guideline for the Evaluation and Management of Chronic Kidney Disease. Kidney Int. **2024**; 105(4S):S117-S314. https://doi.org/10.1016/j.kint.2023.10.018.
17. Visseren FLJ, Mach F, Smulders YM, Carballo D, Koskinas KC, Bäck M, et al; ESC Scientific Document Group. 2021 ESC Guidelines on cardiovascular disease prevention in clinical practice. Developed by the Task Force for cardiovascular disease prevention in clinical practice with representatives of the European Society of Cardiology and 12 medical societies. With the special contribution of the European Association of Preventive Cardiology (EAPC). Eur Heart J. **2021**; 42:3227–337. https://doi.org/10.1093/eurheartj/ehab484.
18. Ndumele CE, Rangaswami J, Chow SL, Neeland IJ, Tuttle KR, Khan SS, et al.; American Heart Association. Cardiovascular-kidney-metabolic health: A Presidential Advisory from the American Heart Association. Circulation. **2023**; 148(20):1606-1635. https://doi.org/10.1161/CIR.0000000000001184.

Table S2. Anthropometric parameters of populations with AO, high-WtHR and EA

	With AO		With high-WtHR		With EA		*p*-value (difference in means)		
	Mean (SD)	Median (IQR)	Mean (SD)	Median (IQR)	Mean (SD)	Median (IQR)	AO *vs.* high-WtHR	AO *vs.* EA	High-WtHR *vs.* EA
Weight cm	83.5 (15.3)*	81.8 (71.7–92.5)	84.0 (15.4)	82.4 (73.0–93.3)	78.6 (14.9)	77.0 (68.0–83.0)	0.239	< 0.001	< 0.001
Height cm	164.8 (10.1)	162.0 (155.0–170.0)	161.3 (9.8)	160.5 (154.0–168.0)	163.1 (9.8)	163.0 (156.0–170.0)	< 0.001	< 0.001	< 0.001
BMI kg/m^2	31.3 (4.6)	30.7 (28.2–33.7)	32.1 (4.5)	31.5 (29.1–34.5)	29.5 (4.5)	28.7 (26.3–31.7)	< 0.001	< 0.001	< 0.001
WC cm	104.7 (10.3)	104.0 (97.0–111.0)	107.1 (9.5)	106.0 (100.8–112.0)	98.1 (12.5)	98.0 (89.0–106.0)	< 0.001	< 0.001	< 0.001
WtHR	0.64 (0.06)	0.64 (0.60–0.68)	0.66 (0.06)	0.65 (0.62–0.69)	0.60 (0.08)	0.60 (0.55–0.65)	< 0.001	< 0.001	< 0.001
CUN-BAE %	40.6 (7.0)	41.1 (34.8–45.9)	41.0 (7.3)	41.7 (34.3–46.8)	37.8 (7.4)	37.7 (34.5–43.4)	0.044	< 0.001	< 0.001

AO: abdominal obesity; BMI: body mass index; CI: confidence interval; CUN-BAE: according to its acronym in Spanish, *Clínica Universitaria de Navarra* - Body Adiposity Estimator; IQR: interquartile range; SD: standard deviation. WC: Waist circumference; WtHR: waist-to-height ratio

Figure S2. A) Relationship between age and WC in people with AO in men and women

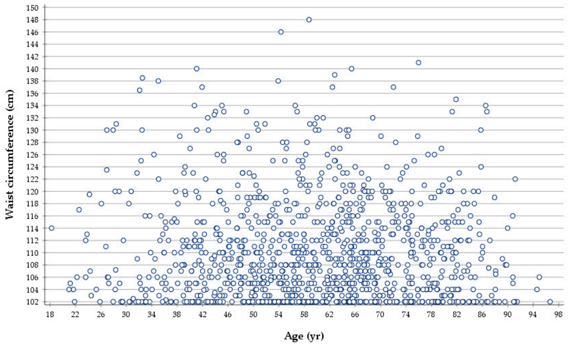

Men ρ = -0.028 (p= 0.353)

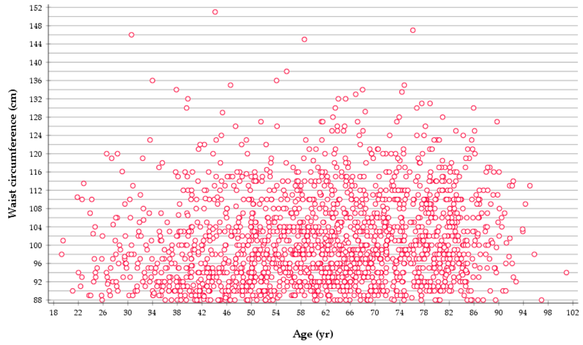

Women ρ = 0.142 (p < 0.001)

B) Relationship between age and WtHR in people with high-WtHR in men and women

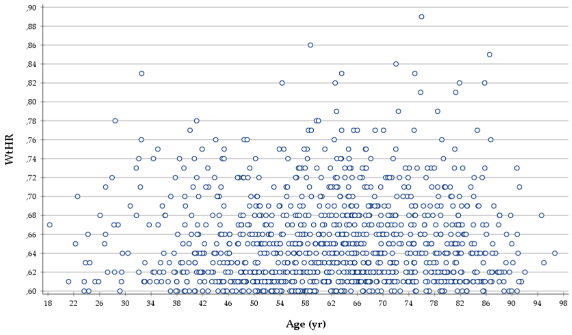

Men ρ= 0.142 (p < 0.001)

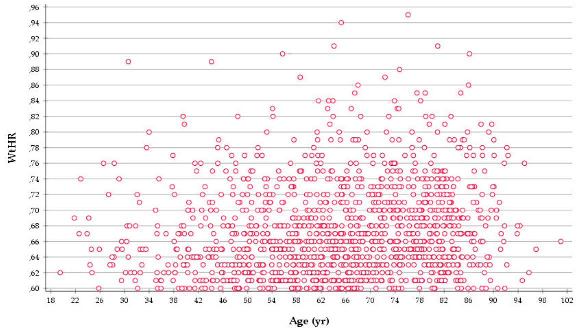

Women ρ = 0.038 (p= 0.217)

C) Relationship between age and CUN-BAE in people with EA in men and women

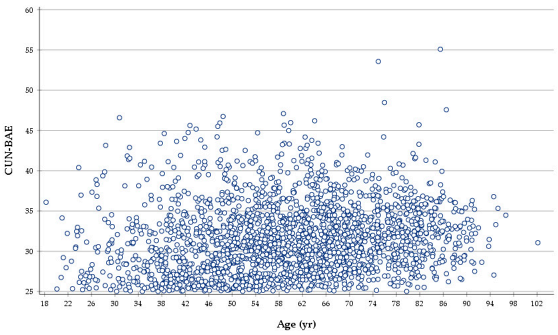

Men ρ = 0.155 (p < 0.001)

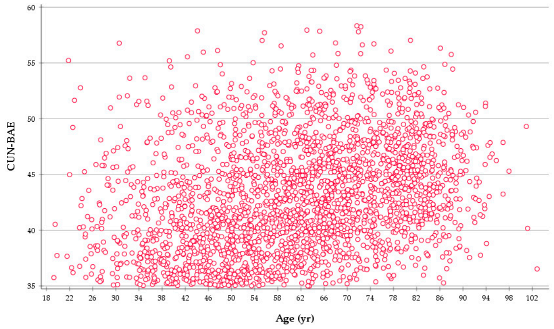

Women ρ = 0.301 (p < 0.001)

ρ: Strength of the correlations according to Pearson's coefficient: null (0.0 < 0.1), low (0.1 < 0.3), medium (0.3 < 0.5), high (0.5 < 0.7) and very high (0.7 < 1.0). AO: abdominal obesity; CUN-BAE: according to its acronym in Spanish, *Clínica Universitaria de Navarra* - Body Adiposity Estimator; EA: excess adiposity; WC: waist circumference; WtHR: waist-to-height ratio

Table S3. Correlations among anthropometric parameters

		WC	WtHR
Overall population	WC	1	0.919**
	CUN-BAE	0.445**	0.656**
Population with abdominal obesity	WC	1	0.795**
	CUN-BAE	0.066**	0.473**
Population with high-WtHR	WC	1	0.742**
	CUN-BAE	0.092**	0.516**
Population with excess adiposity	WC	1	0.883**
	CUN-BAE	0.235**	0.522**

** The correlation is significant at the 0.001 level (two-tailed). Strength of the correlations according to Pearson's ρ coefficient: null (0.0 < 0.1), low (0.1 < 0.3), medium (0.3 < 0.5), high (0.5 < 0.7) and very high (0.7 < 1.0). CUN-BAE: according to its acronym in Spanish, *Clínica Universitaria de Navarra* - Body Adiposity Estimator; WC: waist circumference; WtHR: waist-to-height ratio

Table S4. Comorbidities and medical conditions in populations with and without AO, high-WtHR, and EA

	With AO N= 2922 No. (%)	Without AO N= 3666 No. (%)	*p*	OR (95% CI)	With high-WtHR N= 2361 No. (%)	Without high -WtHR N= 4227 No. (%)	*p*	OR (95% CI)	With EA N= 4832 No. (%)	Without EA N= 1756 No. (%)	*p*	OR (95% CI)
Current smoking	526 (18.0)	900 (24.6)	<0.001	0.7 (0.6–0.8)	404 (17.1)	1022 (24.2)	<0.001	0.7 (0.6–0.7)	923 (19.1)	503 (28.6)	<0.001	0.6 (0.5–0.7)
Alcoholism	262 (9.0)	348 (9.5)	0.464	0.9 (0.8–1.1)	227 (9.6)	383 (9.1)	0.457	1.1 (0.9–1.3)	457 (9.5)	153 (8.7)	0.356	1.1 (0.9–1.3)
Physical inactivity	1581 (54.1)	1498 (40.9)	<0.001	1.7 (1.6–1.9)	1310 (55.5)	1769 (41.9)	<0.001	1.7 (1.6–1.9)	2413 (49.9)	666 (37.9)	<0.001	1.6 (1.5–1.8)
Overweight	1102 (37.7)	1414 (38.6)	0.477	1.0 (0.9–1.1)	754 (31.9)	1762 (41.7)	<0.001	0.7 (0.6–0.7)	2391 (49.5)	125 (7.1)	<0.001	12.8 (10.6–15.5)
Obesity	1675 (57.3)	158 (4.3)	<0.001	29.8 (25.0–35.5)	1554 (65.8)	279 (6.6)	<0.001	27.2 (23.5–31.6)	1833 (37.9)	0 (0.0)	NE	NE
AO	2922 (100.0)	0 (0.0)	NE	NE	2196 (93.0)	726 (17.2)	<0.001	64.2 (53.8–76.6)	2860 (59.2)	62 (3.5)	<0.001	39.6 (30.6–51.4)
High WtHR	2813 (96.3)	883 (24.1)	<0.001	81.3 (66.2–99.9)	2361 (100.0)	0 (0.0)	NE	NE	2344 (48.5)	17 (1.0)	<0.001	96.4 (59.6–155.9)
CUN-BAE EA	2860 (97.9)	1972 (53.8)	<0.001	39.6 (30.6–51.4)	2344 (99.3)	2488 (58.9)	<0.001	96.4 (59.6–155.9)	4832 (100.0)	0 (0.0)	NE	NE
Prediabetes	828 (28.3)	621 (16.9)	<0.001	1.9 (1.7–2.2)	722 (30.6)	727 (17.2)	<0.001	2.1 (1.9–2.4)	1273 (26.3)	176 (10.0)	<0.001	3.2 (2.7–3.8)
Diabetes	677 (23.2)	359 (9.8)	<0.001	2.8 (2.4–3.2)	639 (27.1)	397 (9.4)	<0.001	3.6 (3.1–4.1)	966 (20.0)	70 (4.0)	<0.001	6.0 (4.9–7.7)
Hypertension	1674 (57.3)	1012 (27.6)	<0.001	3.5 (3.2–3.9)	1518 (64.3)	1168 (27.6)	<0.001	4.7 (4.2–5.3)	2524 (52.2)	162 (9.2)	<0.001	10.8 (9.1–12.8)
Hypercholesterolaemia	2105 (72.0)	1996 (54.4)	<0.001	2.2 (1.9–2.4)	1755 (74.3)	2377 (56.2)	<0.001	2.3 (2.0–2.5)	3417 (70.7)	715 (40.7)	<0.001	3.5 (3.1–3.9)
Low HDL-C	1051 (36.0)	768 (20.9)	<0.001	2.1 (1.9–2.4)	872 (36.9)	947 (22.4)	<0.001	2.0 (1.8–2.3)	1529 (31.6)	290 (16.5)	<0.001	2.3 (2.0–2.7)
Hypertriglyceridaemia	1110 (38.0)	837 (22.8)	<0.001	2.1 (1.9–2.3)	949 (40.2)	998 (23.6)	<0.001	2.2 (2.0–2.4)	1687 (34.9)	260 (14.8)	<0.001	3.1 (2.7–3.6)
Atherogenic dyslipidaemia	596 (20.4)	345 (9.4)	<0.001	2.5 (2.1–2.8)	508 (21.5)	433 (10.2)	<0.001	2.4 (2.1–2.8)	850 (17.6)	91 (5.2)	<0.001	3.9 (3.1–4.9)
Metabolic syndrome	2036 (69.7)	815 (22.2)	<0.001	8.0 (7.2–9.0)	1748 (74.0)	1103 (26.1)	<0.001	8.1 (7.2–9.1)	2720 (56.3)	131 (7.5)	<0.001	16.0 (13.3–19.3)
FLI ≥ 60 [a,b,c]	1759 (65.4)	391 (11.4)	<0.001	14.6 (12.8–16.7)	1624 (75.1)	526 (13.3)	<0.001	19.6 (17.1–22.4)	2105 (47.1)	45 (2.7)	<0.001	31.5 (23.3–42.6)
CHD	187 (6.4)	134 (3.7)	<0.001	1.8 (1.4–2.3)	187 (7.9)	134 (3.2)	<0.001	2.6 (2.1–3.3)	302 (6.3)	19 (1.1)	<0.001	6.1 (3.8–9.7)
Stroke	149 (5.1)	101 (2.8)	<0.001	1.9 (1.5–2.5)	141 (6.0)	109 (2.6)	<0.001	2.4 (1.9–3.1)	234 (4.8)	16 (0.9)	<0.001	5.5 (3.3–9.2)
PAD	84 (2.9)	66 (1.8)	0.004	1.6 (1.2–2.2)	82 (3.5)	68 (1.6)	<0.001	2.2 (1.6–3.0)	138 (2.9)	12 (0.7)	<0.001	4.3 (2.4–7.7)
ASCVD	355 (12.1)	260 (7.1)	<0.001	1.8 (1.5–2.1)	348 (14.7)	267 (6.3)	<0.001	2.6 (2.2–3.0)	573 (11.9)	42 (2.4)	<0.001	5.5 (4.0–7.5)
Heart failure	113 (3.9)	71 (1.9)	<0.001	2.0 (1.5–2.8)	109 (4.6)	75 (1.8)	<0.001	2.7 (2.0–3.6)	175 (3.6)	9 (0.5)	<0.001	7.3 (3.7–14.3)
Atrial fibrillation	164 (5.6)	86 (2.3)	<0.001	2.5 (1.9–3.2)	155 (6.6)	95 (2.2)	<0.001	3.1 (2.4–4.0)	239 (4.9)	11 (0.6)	<0.001	8.3 (4.5–15.1)
CVD	494 (16.9)	337 (9.2)	<0.001	2.0 (1.7–2.3)	477 (20.2)	354 (8.4)	<0.001	2.8 (2.4–3.2)	777 (16.1)	54 (3.1)	<0.001	6.0 (4.6–8.0)
Erectile dysfunction [d,e,f]	232 (21.1)	272 (15.1)	<0.001	1.5 (1.2–1.8)	246 (23.0)	258 (14.1)	<0.001	1.8 (1.5–2.2)	472 (21.6)	32 (4.5)	<0.001	5.9 (4.1–8.5)
Hyperuricaemia [g,h,i]	438 (15.2)	302 (8.4)	<0.001	2.0 (1.7–2.3)	406 (17.5)	334 (8.0)	<0.001	2.4 (2.1–2.8)	665 (14.0)	75 (4.3)	<0.001	3.6. (2.8–4.6)

Albuminuria	231 (7.9)	163 (4.4)	<0.001	1.8 (1.5–2.3)	225 (9.5)	169 (4.0)	<0.001	2.5 (2.1–3.1)	358 (7.4)	36 (2.1)	<0.001	3.8 (2.7–5.4)
Low eGFR	328 (11.2)	196 (5.3)	<0.001	2.2 (1.9–2.7)	318 (13.5)	206 (4.9)	<0.001	3.0 (2.5–3.6)	504 (10.4)	20 (1.1)	<0.001	10.1 (6.4–15.9)
CKD	474 (16.2)	282 (7.7)	<0.001	2.3 (2.0–2.7)	451 (19.1)	305 (7.2)	<0.001	3.0 (2.6–3.5)	709 (14.7)	47 (2.7)	<0.001	6.3 (4.6–8.4)
Low risk CKD	2448 (83.8)	3384 (92.3)	<0.001	0.9 (0.9–0.9)	1910 (80.9)	3922 (92.8)	<0.001	0.9 (0.9–0.9)	4123 (85.3)	1709 (97.3)	<0.001	0.9 (0.9–0.9)
Moderate risk CKD	308 (10.5)	182 (5.0)	<0.001	2.1 (1.8–2.5)	281 (11.9)	209 (4.9)	<0.001	2.4 (2.0–2.9)	456 (9.4)	34 (1.9)	<0.001	4.9 (3.5–6.9)
High risk CKD	112 (3.8)	52 (1.4)	<0.001	2.7 (2.0–3.7)	109 (4.6)	55 (1.3)	<0.001	3.5 (2.6–4.9)	156 (3.2)	8 (0.5)	<0.001	7.1 (3.5–14.4)
Moderate- to high-risk CKD	420 (14.4)	234 (6.4)	<0.001	2.3 (1.9–2.6)	390 (16.5)	264 (6.2)	<0.001	2.6 (2.3–3.1)	612 (12.7)	42 (2.4)	<0.001	5.3 (3.9–7.2)
Very high risk CKD	54 (1.8)	48 (1.3)	0.078	1.4 (1.0–2.1)	61 (2.6)	41 (1.0)	<0.001	2.7 (1.8–3.9)	97 (2.0)	5 (0.3)	<0.001	7.1 (2.9–17.3)
Low CVR	454 (15.5)	1691 (46.1)	<0.001	0.2 (0.2–0.2)	221 (9.4)	1924 (45.5)	<0.001	0.1 (0.1–0.1)	885 (18.3)	1260 (71.8)	<0.001	0.1 (0.1–0.1)
Moderate CVR	694 (23.8)	685 (18.7)	<0.001	1.4 (1.2–1.5)	533 (22.6)	846 (20.0)	0.014	1.2 (1.0–1.3)	1147 (23.7)	232 (13.2)	<0.001	2.0 (1.8–2.4)
High CVR	539 (18.4)	484 (13.2)	<0.001	1.5 (1.3–1.7)	421 (17.8)	602 (14.2)	0.001	1.3 (1.1–1.5)	872 (18.0)	151 (8.6)	<0.001	2.4 (2.0–2.8)
Very high CVR	1235 (42.3)	806 (22.0)	<0.001	2.6 (2.3–2.9)	1186 (50.2)	855 (20.2)	<0.001	4.0 (3.6–4.4)	1928 (39.9)	113 (6.4)	<0.001	9.7 (7.9–11.8)

AO: abdominal obesity; WtHR: waist-to-height ratio; EA: excess adiposity. No. (%): cases number (percentage); CI: confidence interval; OR: odds ratio; *p*: *p*-value of the difference in percentage; NE: not estimable.

ASCVD: atherosclerotic cardiovascular disease; CHD: coronary heart disease; CKD: chronic kidney disease; CUN-BAE: according to its acronym in Spanish, *Clínica Universitaria de Navarra* - Body Adiposity Estimator; CVD: cardiovascular diseases; CVR: cardiovascular risk; eGFR: estimated glomerular filtration rate; FLI: fatty liver index; HDL-C: high-density lipoprotein cholesterol; PAD: peripheral arterial disease. The definitions of comorbidities or medical conditions are shown in Table S1 (Suppl. Material).

[a] N= 2690 with AO, 3418 without AO; [b] N= 2163 with high-WtHR, 3945 without high -WtHR: [c] N= 4470 with AE, 1638 without AE

[d] N= 1102 with AO, 1802 without AO; [e] N= 1069 with high-WtHR, 1835 without high-WtHR; [f] N= 2189 with AE, 715 without AE

[g] N= 2878 with AO, 3611 without AO; [h] N= 2319 with high-WtHR, 4170 without high -WtHR; [i] N= 4755 with AE, 1734 without AE

Table S5. Multivariate analysis of factors and medical conditions for AO, high-WtHR, and EA

	AO				High-WtHR				EA			
	Wald	β [a]	OR Exp(β) [b]	*p* [c]	Wald	β [a]	OR Exp(β) [b]	*p* [c]	Wald	β [a]	OR Exp(β) [b]	*p* [c]
Hypertension	213.6	0.89 (0.06)	2.43 (2.16-2.74)	<0.001	312.4	1.11 (0.06)	3.03 (2.68-3.43)	<0.001	342.2	1.81 (0.10)	6.13 (5.06-7.42)	<0.001
Diabetes	53.9	0.61 (0.08)	1.84 (1.57-2.17)	<0.001	109.1	0.88 (0.08)	2.41 (2.04-2.84)	<0.001	48.3	0.98 (0.14)	2.65 (2.02-3.49)	<0.001
Prediabetes	72.3	0.58 (0.07)	1.78 (1.56-2.03)	<0.001	114.4	0.75 (0.07)	2.12 (1.84-2.43)	<0.001	93.9	0.92 (0.10)	2.50 (2.08-3.01)	<0.001
Low HDL-C	75.3	0.55 (0.06)	1.74 (1.53-1.97)	<0.001	46.9	0.46 (0.07)	1.58 (1.38-1.80)	<0.001	54.4	0.61 (0.08)	1.85 (1.57-2.18)	<0.001
Hypercholesterolaemia	38.9	0.38 (0.06)	1.47 (1.30-1.65)	<0.001	19.4	0.29 (0.07)	1.34 (1.18-1.53)	<0.001	123.5	0.76 (0.07)	2.13 (1.86-2.43)	<0.001
Hypertriglyceridaemia	6.1	0.16 (0.07)	1.17 (1.03-1.33)	0.014	7.1	0.18 (0.07)	1.20 (1.05-1.37)	0.008	11.9	0.30 (0.09)	1.36 (1.14-1.61)	0.001
Physical inactivity	86.4	0.51 (0.05)	1.66 (1.49-1.84)	<0.001	84.4	0.53 (0.06)	1.71 (1.52-1.91)	<0.001	46.5	0.45 (0.07)	1.56 (1.37-1.77)	<0.001
Hyperuricaemia	5.2	0.20 (0.09)	1.22 (1.03-1.45)	0.022	17.4	0.37 (0.09)	1.45 (1.22-1.72)	<0.001	13.0	0.50 (0.14)	1.65 (1.26-2.17)	<0.001
CKD	2.0	0.13 (0.09)	1.14 (0.95-1.36)	0.152	10.0	0.29 (0.09)	1.33 (1.12-1.59)	0.002	18.9	0.74 (0.17)	2.09 (1.50-2.92)	<0.001

AO: abdominal obesity; CKD: chronic kidney disease; EA: excess adiposity; HDL-C: high-density lipoprotein cholesterol; WtHR: waist-to-height ratio. Definitions of the clinical conditions are shown in Table S1 (Suppl. Material).

[a] β coefficient (± deviation); [b] OR Exp (β): odds-ratio (95% confidence interval); [c] *p*: *p*-value of Wald test with one degree of freedom
